# Push-pull competition between bottom-up and top-down auditory attention to natural soundscapes

**DOI:** 10.7554/eLife.52984

**Published:** 2020-03-20

**Authors:** Nicholas Huang, Mounya Elhilali

**Affiliations:** 1 Laboratory for Computational Audio Perception, Department of Electrical Engineering, Johns Hopkins UniversityBaltimoreUnited States; Peking UniversityChina; Carnegie Mellon UniversityUnited States

**Keywords:** auditory, Attention, top-down, bottom-up, salience, Human

## Abstract

In everyday social environments, demands on attentional resources dynamically shift to balance our attention to targets of interest while alerting us to important objects in our surrounds. The current study uses electroencephalography to explore how the push-pull interaction between top-down and bottom-up attention manifests itself in dynamic auditory scenes. Using natural soundscapes as distractors while subjects attend to a controlled rhythmic sound sequence, we find that salient events in background scenes significantly suppress phase-locking and gamma responses to the attended sequence, countering enhancement effects observed for attended targets. In line with a hypothesis of limited attentional resources, the modulation of neural activity by bottom-up attention is graded by degree of salience of ambient events. The study also provides insights into the interplay between endogenous and exogenous attention during natural soundscapes, with both forms of attention engaging a common fronto-parietal network at different time lags.

## eLife digest

When walking into a busy restaurant or café, our sense of hearing is bombarded with different sounds that our brain has to sort through to make sense of our surroundings. Our brain has to balance the desire to focus our attention on sounds we choose to listen to (such as the friend we are having a conversation with) and sounds that attract our attention (such as the sound of someone else’s phone ringing). Without the ability to be distracted, we might miss a noise that may or may not be crucial to our survival, like the engine roar of an approaching vehicle or a ping notifying us of an incoming email. However, it remains unclear what happens in our brains that enables us to shift our attention to background sounds.

To investigate this further, Huang and Elhilali asked 81 participants to focus their attention on a repeating sound while being exposed to background noises from everyday life, such as sounds from a busy café. The experiment showed that when a more noticeable sound happened in the background, such as a loud voice, the participants were more likely to lose attention on their task and miss changes in the tone of the repeating sound.

Huang and Elhilali then measured the brain activity of 12 participants as they counted the number of altered tones in a sequence of sounds, again with noise in the background. This revealed that brain waves synchronized with tones that the participants were concentrating on. However, once there was a noticeable event in the background, this tone synchronization was reduced and the brain waves aligned with the background noise. Huang and Elhilali found that distracting noises in the background activate the same region of the brain as sounds we choose to listen to. This demonstrates how background sounds are able to re-direct our attention.

These results are consistent with the idea that we have a limited capacity for attention, and that new sensory information can divert brain activity. Having a better understanding of how these processes work could help develop better communication aids for people with impaired hearing, and improve software for interpreting sounds with a noisy background.

## Introduction

Attention is a selection mechanism that deploys our limited neural resources to the most relevant stimuli in the environment. Without such a process, the sights and sounds of everyday life would overwhelm our senses. Filtering out important information from our surroundings puts a constant demand on the cognitive system given the dynamic nature of everyday scenes. On the one hand, we attend to sounds, sights and smells that we choose based on what matches our behavioral goals and contextual expectations. At the same time, we have to balance perception of salient events and objects that we need to be alerted to both for survival as well as awareness of our ever changing surrounds. These various factors guide our attentional resources to dynamically shift in order to shape the representation of sensory information based on its behavioral relevance, and ultimately influence how we perceive the world around us.

How does the brain manage its executive attentional resources faced with these dynamic demands? Studies of voluntary (‘top-down’ or endogenous) attention have shown that cognitive feedback modulates the encoding of sensory cues in order to improve the signal-to-noise ratio of attended targets relative to irrelevant maskers or other objects in the environment (Baluch and Itti, 2011; Desimone and Duncan, 1995; Senkowski et al., 2005). Whether attending to someone’s voice amidst a crowd, or spotting that person in a busy street, or even identifying a particular smell among others, changes to neural encoding due to attention have been reported across all sensory modalities including auditory, visual, olfactory and somatosensory systems and appear to operate across multiple neural scales (Corbetta and Shulman, 2002; Zelano et al., 2005; Bauer et al., 2006; Knudsen, 2007). Response profiles of individual neurons, entire sensory circuits, and cross brain regions are modulated by attentional feedback to produce neural responses that reflect not only the physical properties of the stimulus but also the behavioral state and reward expectations of the system. Some of the hallmarks of selective attention are enhanced neural encoding in sensory cortex of attended features and dynamics (e.g. envelope of an attended speaker; Mazziotta et al., 2001) as well as recruitment of dorsal fronto-parietal circuits mediated by boosted gamma oscillatory activity (Fries, 2009; Baluch and Itti, 2011).

In contrast, our understanding of the effects of involuntary (‘bottom-up’ or exogenous) attention has been mostly led by work in vision. There is a well established link between perceptual attributes of salience and their influence on attention deployment in visual scenes (Borji et al., 2013; Treue, 2003; Wolfe and Horowitz, 2004). Studies of visual salience greatly benefited from natural behaviors such as eye gaze which facilitate tracking subjects’ attentional focus and allow the use of rich and naturalistic stimuli including still pictures and videos (Borji, 2015; Carmi and Itti, 2006; Marius 't Hart et al., 2009). In parallel, exploration of brain networks implicated in visual salience revealed engagement of both subcortical and cortical circuits that balance the sensory conspicuity of a visual scene with more task-related information to shape attentional orienting to salient visual objects (Veale et al., 2016). Bottom-up attention also engages ventral fronto-parietal circuits that orient subjects’ focus to salient stimuli outside the spotlight of voluntary attention (Corbetta and Shulman, 2002; Fox et al., 2006; Asplund et al., 2010).

By comparison, the study of auditory bottom-up attention has proven more challenging owing to the difficulty of properly defining behavioral metrics that determine when attention is captured by a salient sound. Pupilometry has been explored in auditory salience studies with recent evidence suggesting a correlation between stimulus salience and pupil size (Liao et al., 2016; Wang et al., 2014; Zhao et al., 2019). Still, there are various aspects of this correlation that remain ill understood, with some evidence suggesting that a large component of the pupillary response is driven by the sound’s loudness and its local context rather than a full account of its perceptual salience (Liao et al., 2017; Huang and Elhilali, 2017). In parallel, a large body of work has focused on deviance detection in auditory sequences, and has established neural markers associated with deviant or rare events. An example of such a response is mismatch negativity (MMN) which can be elicited pre-attentively, though this response is modulated by attention and associated with behavioral measures of distraction (Ririe et al., 2017; Näätänen et al., 2007). By using sequences with deviant tokens or snippets, studies of novelty processing are able to better control the acoustic parameters of the stimulus and the precise occurrence and nature of salient events. Other studies have extended oddball designs using richer sound structures including musical sequences or noise patterns, but still piecing together sound tokens to control presence of transient salient events in the acoustic signal (Duangudom and Anderson, 2013; Kayser et al., 2005; Kaya and Elhilali, 2014; Tordini et al., 2015). Nonetheless, this structure falls short of the natural intricacies of realistic sounds in everyday environments where salience can take on more nuanced manifestations. Similar to established results in vision, use of natural soundscapes could not only extend results observed with simpler oddball sequences; but also shed light on the privileged processing status of social signals that reflect attentional capture in everyday life (Doherty et al., 2017).

In the current work, we explore dynamic deployment of attention using an unconstrained dataset of natural sounds. The collection includes a variety of contents and compositions, and spans samples from everyday situations taken from public databases such as YouTube and Freesound. It covers various settings such as a busy cafeteria, a ballgame at the stadium, a concert in a symphony hall, a dog park, and a protest in the streets. Concurrent with these everyday sounds, subjects’ attention is directed towards a controlled tone sequence where we examine effects of top-down attention to this rhythmic sequence as well as intermittent switches of attention elicited by salient events in background scenes. This paradigm tackles three key limitations in our understanding of the dynamic deployment of attentional resources in complex auditory scenes. First, the study investigates the relationship between auditory salience and bottom-up attentional capture beyond deviance detection paradigms. The stimulus probes how distracting events in a background scene organically compete for attentional resources already deployed toward a dynamic sound sequence. Unlike clearly defined ‘deviants’ typically used in oddball paradigms, the attention-grabbing nature of salient events in natural soundscapes is more behaviorally and cognitively nuanced and exhibits a wide range of dynamics both in strength and buildup. A salient event in a complex scene can vary from a momentary transient event (e.g. a phone ringing in an auditorium) to a gradually dynamic sound (e.g. a distinct voice steadily emerging from the cacophony in a busy cafeteria). Here, we are interested in probing whether this natural and nuanced capture of attention induces equally profound effects on brain responses as previously reported from top-down, task-related attention. The study leverages the organic nature of competition between bottom-up and top-down attention in natural soundscapes to not only test the hypothesis of limited resources shared between the two modes of attention, but also engagement of distinct but overlapping brain networks (Corbetta and Shulman, 2002; Salmi et al., 2009).

Second, employing dynamic scenes allows us to focus our analysis beyond event-related potentials (ERPs) which require precisely-aligned event onsets, hence often limiting paradigms to oddball designs or intermittent distractors. In the current study, the use of continuous scenes is anchored against a rhythmic attended sequence which provides a reference for temporal alignment of competing attentional states throughout the experiment. Third, while the study sheds lights on neural markers of auditory salience, it does so *relative to* a competing sequence in a controlled attentional task. As such, it balances the dichotomy often found in studies of auditory salience using either distraction or detection paradigms. A number of studies probe bottom-up attention using irrelevant stimuli presented to the subjects without necessarily competing for their attentional focus; or where subjects are informed or learn their value (see Lavie, 2005). Here, we are interested in characterizing the dynamic effect of salient distractors on the encoding of attended targets. Ultimately, the current study aims to determine how well we can predict the existence of attention-grabbing events while subjects are engaged in a competing task.

## Results

**Figure 1.**
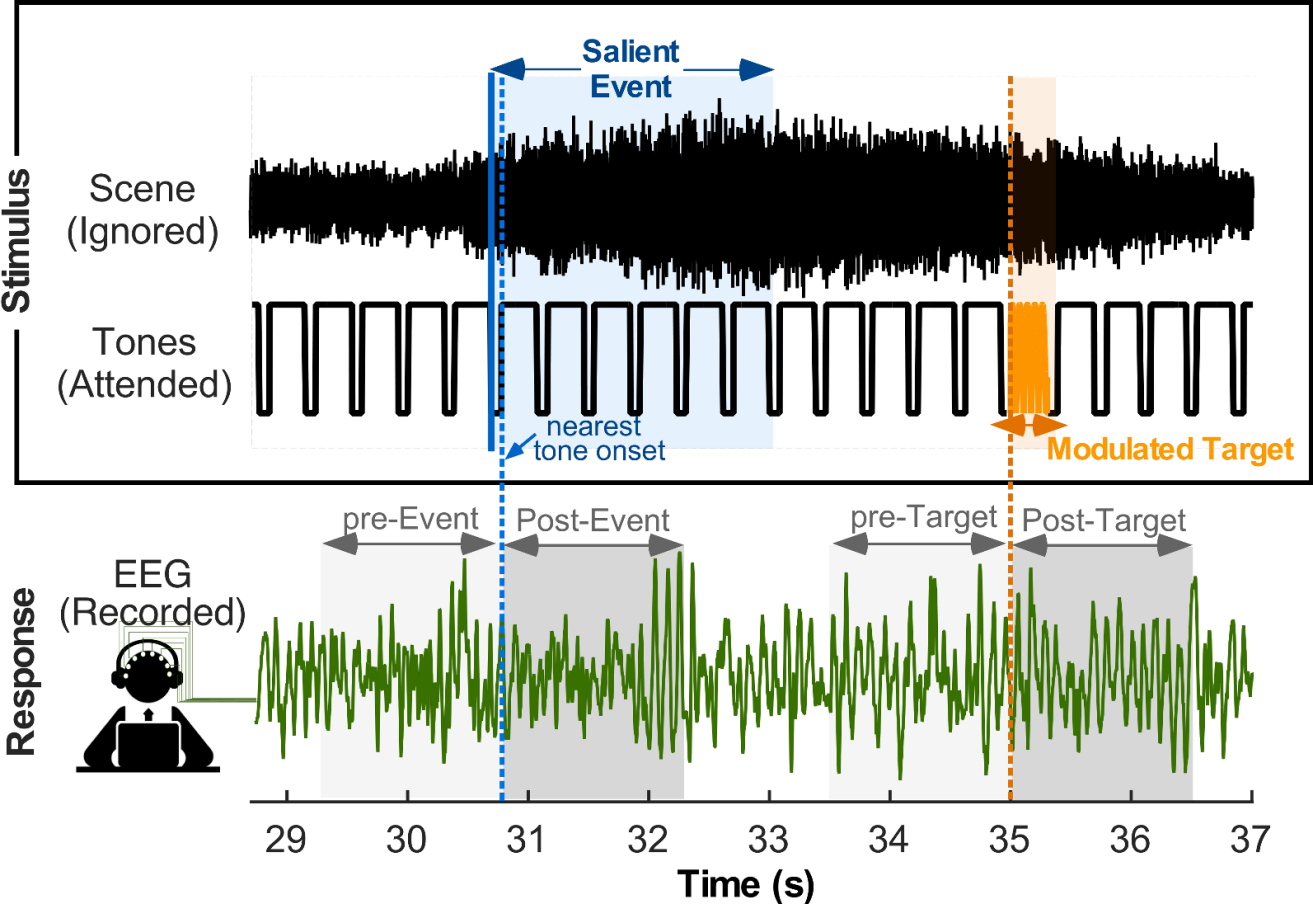
Stimulus paradigm during EEG recording. Listeners are presented with two concurrent sounds in each each trial: (top stimulus) A recording of a natural audio clip, which subjects are asked to ignore; and (bottom stimulus) a rhythmic tone sequence, which subjects pay attention to and detect presence of occasional modulated tones (shown in orange). A segment of one trial neural recording is shown in the bottom. Analyses focus on changes in neural responses due to presence of salient events in the ambient scene or target tones in the attended scene.

Listeners perform an amplitude-modulation (AM) detection task by attending to a tone sequence and indicating presence of intermittent modulated target tones (orange note in Figure 1). Concurrently, a busy acoustic scene is presented in the background and subjects are asked to completely ignore it. Background scenes are taken from the JHU DNSS (Dichotic Natural Salience Soundscapes) database for which behavioral estimates of salience timing and strength have been previously collected (Huang and Elhilali, 2017) (see Materials and methods for details). In a first experiment, easy and hard AM detection tasks are interleaved in experimental blocks by changing the modulation depth of the target note (easy: 0 dB, hard: −5 dB). As expected, subjects report a higher overall detection accuracy for the easy condition (75.4%) compared to the hard condition (48.2%). Moreover, target detection (in both easy and hard conditions) is disrupted by presence of a salient event in the ignored background scenes; and detection accuracy drops significantly over a period up to a second after onset of the salient event [drop in detection accuracy; hard task, t(62) = −5.25, p=1.96*10^−6^; easy task, t(62) = −5.62, p=4.92*10^−7^]. Salient events attract listeners’ attention away from the task at hand and cause a drop in detection accuracy that is proportional to the salience level of background distractors; especially for high and mid salience events [hard task - high salience event t(62) = −4.97, p=5.57*10^−6^; mid salience event t(62) = −3.70, p=4.54*10^−4^; low salience event t(62) = −0.75, p=0.46; easy task - high salience event t(62) = −4.20, p=8.54*10^−5^; mid salience event t(62) = −2.29, p=0.025; low salience event t(62) = −1.51, p=0.14]. In order to further explore neural underpinnings of changes in the attentional state of listeners, this paradigm is repeated with the easy task while neural activity is measured using Electroencephalography (EEG).

**Figure 2.**
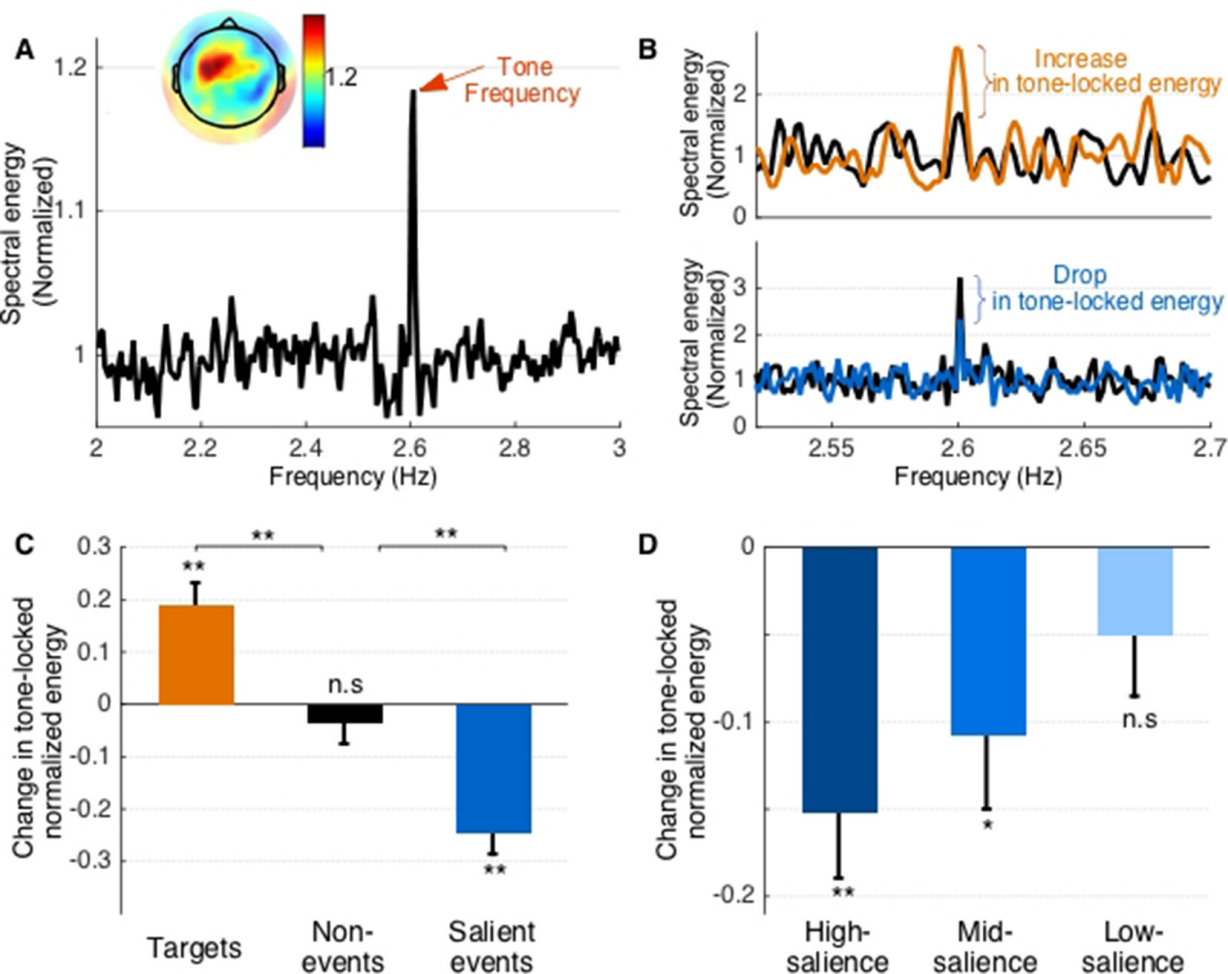
Phase-locking results. (**A**) Spectral density across all stimuli. The peak in energy at the tone presentation frequency is marked by a red arrow. Inset shows average normalized tone-locking energy for individual electrodes. (**B**) Spectral density around target tones (top) and salient events (bottom). Black lines show energy *preceding* the target or event, while colored lines depict energy *following*. Note that target tones are fewer throughout the experiment leading to lower resolution of the spectral profile. (**C**) Change in phase-locking energy across target tones, non-events, and salient events. (**D**) Change in tone-locking energy across high, mid, and low salience events. Error bars depict ±1 SEM.

The attended tone sequence is presented at a regular tempo of 2.6 Hz and induces a strong overall phase-locked response around this frequency despite the concurrent presentation of a natural scene in the background. Figure 2A shows the grand average spectral profile of the neural response observed throughout the experiment. The plot clearly displays a strong energy at 2.6 Hz, with a left-lateralized fronto-central response, consistent with activation of Heschl’s gyrus and conforming to prior observations of precise phase-locking to relatively slow rates in core auditory cortex (Lütkenhöner and Steinsträter, 1998; Liégeois-Chauvel et al., 2004; Stropahl et al., 2018). (Figure 2A, inset).

Taking a closer look at this phase-locked activity aligned to the tone sequence, the response appears to change during the course of each trial, particularly when coinciding with task-specific AM tone targets, as well as when concurring with salient events in the background scene. Phase-locking near modulated-tone targets shows an increase in 2.6 Hz power relative to the average level, reflecting an expected increase in neural power induced by top-down attention (Figure 2B-top). The same phase-locked response is notably reduced when tones coincide with salient events in the background (Figure 2B-bottom - blue curve), indicating diversion of resources away from the attended sequence and potential markers of distraction caused by salient events in the ignored background.

We contrast variability of 2.6 Hz phase-locked energy over 3 windows of interest in each trial: (i) near AM tone targets, (ii) near salient events and (iii) near tones chosen randomly ‘away’ from either targets or salient events and used as control baseline responses. We compare activity in each of these windows relative to a preceding window (e.g. Figure 1, post vs. pre-event interval). Figure 2C shows that phase-locking to 2.6 Hz after target tones increases significantly [t(443)=4.65, p=4.43*10^−6^], whereas it decreases significantly following salient events [t(443)=−5.89, p < 10^−7^], relative to preceding non-target tones. A random sampling of tones away from target tones or salient events does not show any significant variability [t(443)=−0.78, p=0.43, Bayes Factor 0.072] indicating a relatively stable phase-locked power in ‘control’ segments of the experiment away from task-relevant targets or bottom-up background events (2C, middle bar). Compared to each other, the top-down attentional effect due to target tones is significantly different from the inherent variability in phase-locked responses in ‘control’ segments [t(886)=3.81, p=1.48*10^−4^]; while distraction due to salient events induces a decrease in phase-locking that is significantly different from inherent variability in ‘control’ segments [t(886)=−3.58, p=3.66*10^−3^].

Interestingly, this salience-induced decrease is modulated in strength by the level of salience of background events. The decrease in phase-locked energy is strongest for events with a higher level of salience [t(443)=−3.78, p=1.8*10^−4^]. It is also significant for events with mid-level salience [t(443)=−2.57, p=0.01], but marginally reduced though not significant for events with the lowest salience [t(359)=1.33, p=0.20, Bayes Factor BF 0.14] (Figure 2D). A one-way ANOVA did not show a significant difference between the mean suppression at the three salience levels [F(1329)=1.65, p=0.19].

A potential confound to reduced phase-locking due to distraction could be local acoustic variability associated with salient events instead of actual deployment of bottom-up attention that disrupts phase-locking to the attended sequence. While this possibility is unlikely given the significant effect of salient events on behavioral detection of targets, we further reassess loss of phase-locking to the attended rhythm near events by excluding salient events with the highest loudness which could cause energetic masking effects (Moore, 2013). This analysis confirms that phase-locking to 2.6 Hz is still significantly reduced relative to non-event control moments [t(443)=−3.88, p < 10^−3^]. A complementary measure of loudness is also explored by excluding events with the highest energy in one equivalent rectangular bandwidth (ERB) around the tone frequency at 440 Hz (Moore and Glasberg, 1983). Excluding the loudest 25% events by this measure still yields a significant reduction in tone-locking [t(443)=−4.93, p=1.17*10^−6^]. In addition, we analyze acoustic attributes of all salient events in background scenes and compare their acoustic attributes to those of randomly selected intervals in non-salient segments. This comparison assesses whether salient events have unique acoustic attributes that are never observed at other moments in the scene. A Bhattacharyya coefficient -BC- (Kailath, 1967) reveals that salient events share the *same* global acoustic attributes as non-salient moments in the ambient background across a wide range of features (BC for loudness 0.9655, brightness 0.9851, pitch 0.9867, harmonicity .9775 and scale 0.9868). Morever, the significant drop in phase locking is maintained when events are split by strength of low-level acoustic features such as harmonicity or brightness [High Harmonicity, t(443) = −3.75, p=1.97*10^−4^; Low Harmonicity, t(443) = −3.77, p=1.82*10^−4^; High Brightness, t(443) = −4.18, p=3.51*10^−5^; Low Brightness, t(443) = −3.26, p=1.21*10^−3^], further validating that the effect of salience is not solely due to low-level acoustic features.

**Figure 3.**
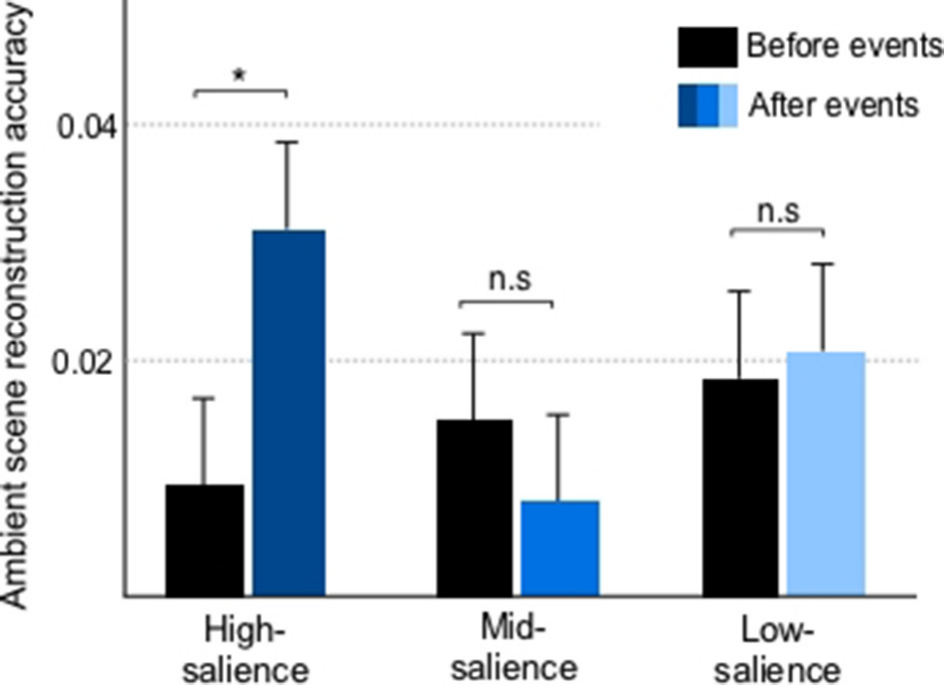
Reconstruction of ignored scene envelopes from neural responses before and after salient events for high, mid and low salience instances. The accuracy quantifies the correlation between neural reconstructions and scene envelopes estimated using ridge regression (see Materials and methods). Error bars depict ±1 SEM.

The reduction of phase-locking to the attended sequences’ rhythm in presence of salient events raises the question whether these ‘attention-grabbing’ instances result in momentary increased neural entrainment to the background scene. While the ambient scene does not contain a steady rate to examine exact phase-locking, its dynamic nature as a natural soundscape allows us to explore the fidelity of encoding of the stimulus envelope before and after salient events. Generally, synchronization of ignored stimuli tends to be greatly suppressed (Ding and Simon, 2012; Fuglsang et al., 2017). Nonetheless, we note a momentary enhancement in decoding accuracy after high salience events compared to a preceding period [paired t-test, t(102) = 2.18, p=0.03] though no such effects are observed in mid [t(113)=−1.09, p=0.28] and low salience [t(107)=0.24, p=0.81] events (Figure 3).

**Figure 4.**
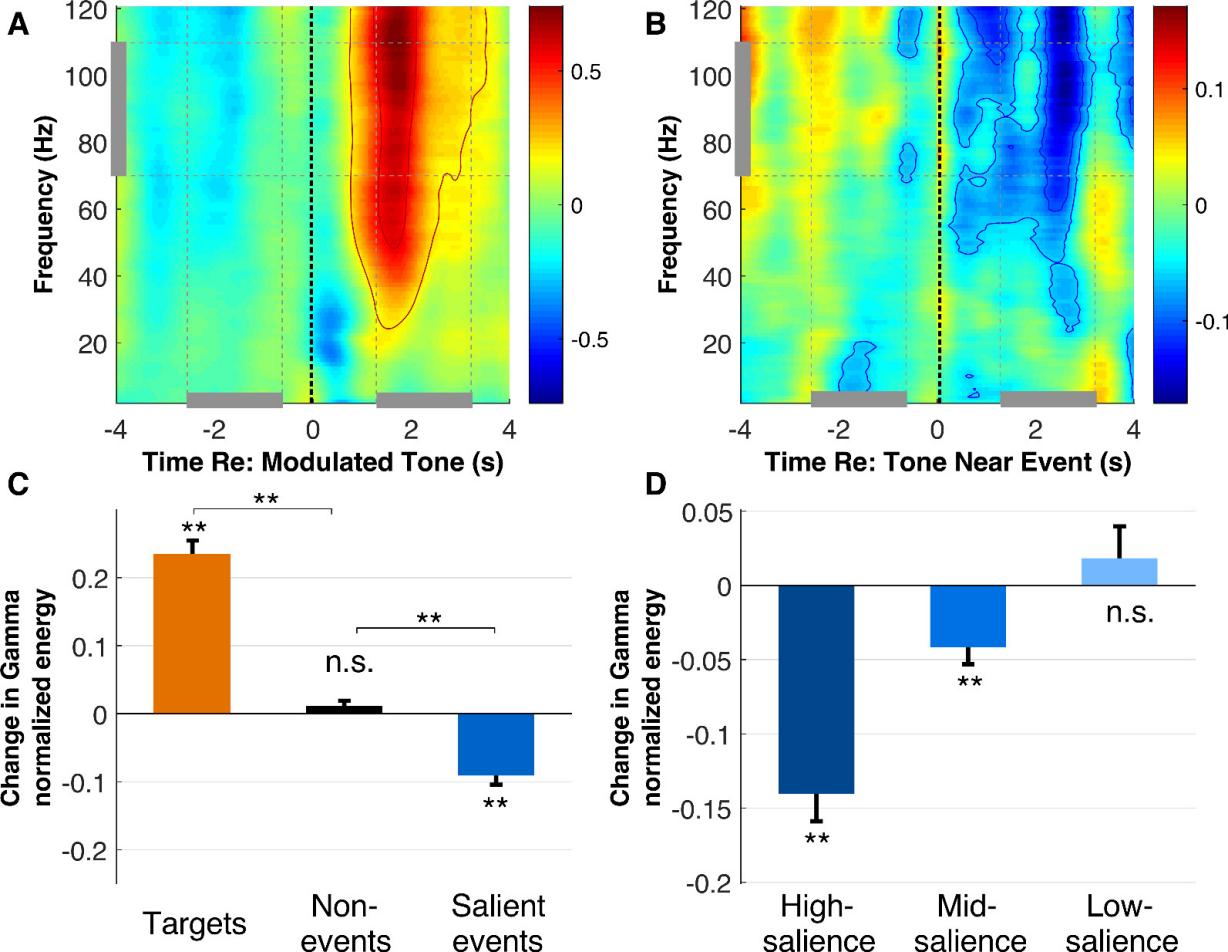
High gamma band energy results. (**A**) Time frequency spectrogram of neural responses aligned to onsets nearest modulated targets, averaged across central and frontal electrodes. Contours depict the highest 80% and 95% of the gamma response. (**B**) Time frequency spectrogram of tones nearest salient events in the background scene. Contours depict the lowest 80% and 95% of the gamma response. (**C**) Change in energy in the high gamma frequency band (70–110 Hz) across target tones, non-events, and salient events relative to a preceding time window. (**D**) Change in high gamma band energy across high, mid, and low salience events. Error bars depict ±1 SEM.

Next, we probe other markers of attentional shift and focus particularly on the Gamma band energy in the neural response (Ray et al., 2008). We contrast spectral profiles of neural responses after target tones, salient events and during ‘control’ tones. Figure 4A depicts a time-frequency profile of neural energy around modulated target tones (0 on the x-axis denotes the start of the target tone). A strong increase in Gamma activity occurs after the onset of target tones and spans a broad spectral bandwidth from 40 to 120 Hz. Figure 4B shows the same time-frequency profile of neural energy relative to attended tones closest to a salient event. The figure clearly shows a decrease in spectral power post-onset of attended tones nearest salient events which is also spectrally broad, though strongest in a high-Gamma range (∼60–120 Hz).

Figure 4C quantifies the variations of Gamma energy relative to targets, salient events, and control tones as compared to a preceding time window. High-Gamma band energy increases significantly following target tones [t(443)=11.5, p < 10^−7^]; while it drops significantly for attended tones near salient events [t(443)=−6.83, p < 10^−7^]. Control non-event segments show no significant variations in Gamma energy [t(443)=1.5, p=0.13, Bayes factor 0.16] confirming a relatively stable Gamma energy throughout the experimental trials overall. The increase in spectral energy around the Gamma band is significantly different in a direct comparison between target and control tones [t(886)=10.3, p < 10^−7^]. Similarly, the decrease in spectral energy around the Gamma band is significantly different when comparing salient events against control tones [t(886)=6.68, p < 10^−7^]. As with the decrease in tone locking, the Gamma band energy drop is more prominent for higher salience events [t(443)=−7.72, p < 10^−7^], is lower but still significant for mid-level salience events [t(443)=−3.64, p=3.02*10^−4^], but not significant for low salience events [t(443)=0.84, p=0.40, Bayes Factor 0.076] (Figure 4D). A one-way ANOVA shows that the three levels of salience strength have significantly different changes in gamma power [F(1329)=20.79, p=1.29*10^−9^], with all levels found to be significantly different from each other based on a post-hoc Tukey test.

Furthermore, the modulation of gamma band energy by both bottom-up and top-down attention is further modulated by subjects’ behavior, quantified using signed error (defined as detected targets minus actual targets - see Materials and methods). Targets in scenes with negative signed error (suggesting that modulated targets were missed due to lower top-down attentional focus) show a smaller increase in gamma power than events in scenes with positive signed error. This difference is significant based on a two-sample t-test [t(886)=−3.96, p=8.06*10^−5^]. Conversely, salient events within negative signed error scenes showed significantly higher increase in gamma than those in positive signed error scenes [t(886)=4.32, p=1.74*10^−5^], suggesting that lower top-down attention indicated higher bottom-up attention, and vice versa. A qualitatively similar result is obtained by grouping subjects’ behavior by error size (absolute error) rather than signed error.

Given this push-pull competition between bottom-up and top-down attentional responses to tones in the attended rhythmic sequence, we examine similarities between neural loci engaged during these different phases of the neural response. Using the Brainstorm software package (Tadel et al., 2011), electrode activations are mapped onto brain surface sources using standardized low resolution brain electromagnetic tomography (sLORETA, see Materials and methods for details). This analysis of localized Gamma activity across cortical voxels examines brain regions *uniquely* engaged while attending to target tones or distracted by a salient event (relative to background activity of control tones).

**Figure 5.**
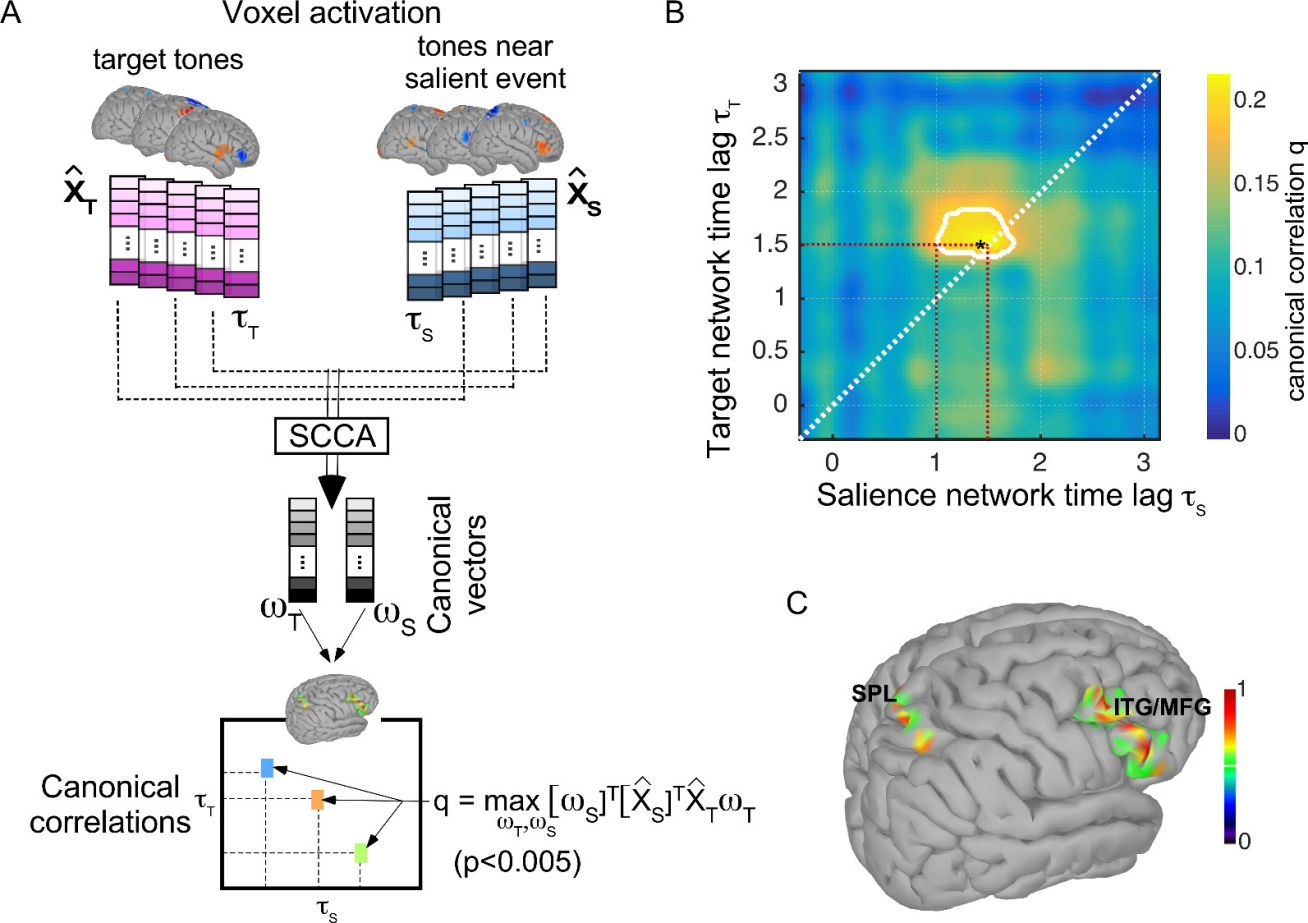
Analysis of overlapping brain networks. (**A**) Sparse canonical correlation analysis (SCCA) is applied to compare top-down (near target) ${\hat{X}}_{T}$ and bottom-up (near salient event) ${\hat{X}}_{S}$ activation maps. Activations at different time lags $\tau_{S}$ and $\tau_{T}$ are compared using SCCA which yields a canonical correlation value q that maximizes the correlation between linear transformations of the original maps; $q=\max_{w_{S},w_{T}}w_{S}^{T}{\hat{X}}_{S}^{T}{\hat{X}}_{T}w_{T}$. A statistical significance (p-value) of the correlation value q is also estimated at each computation lag using a permutation-based approach (see Materials and methods). (**B**) Canonical correlation values q comparing neural activation patterns after tones near salient events (x-axis) and target tones (y-axis). The contour depicts all canonical correlations with statistical significance less than p<0.005. (**C**) Projection of canonical vector (mapping function) that yields maximal correlation between the response after salient events and the response after target tones (at the point shown with an asterisk in panel **B**). The red dashed lines are visual guides to highlight earliest point of observed significant correlation as well as time index of correlation point indicated by an asterisk. The overlap is right-lateralized and primarily located within the superior parietal lobule(SPL), the inferior frontal gyrus(IFG), and the medial frontal gyrus(MFG).

We correlate the topography of these top-down and bottom-up brain voxels using sparse canonical correlation analysis (sCCA) (Roeber et al., 2003; Witten and Tibshirani, 2009; Lin et al., 2013) to estimate multivariate similarity between these brain networks at different time lags (Figure 5A, see Materials and methods for details). Canonical correlation analysis (CCA) is a form of multivariate analysis of correlation where high-dimensional data are compared in order to discover interpretable associations (or correlations) represented as data projections -called canonical vectors- (Uurtio et al., 2018). Imposing sparse constraints on this procedure improves interpretability of these projections by confining these mapping to constrained vectors. We cross-correlate brain activation maps at different time lags, and consider that similar brain networks are engaged if a statistically significant correlation emerges from the canonical analysis. Figure 5B shows that a significant correlation between Gamma activity in brain voxels is observed about 1 s after tone onset, with bottom-up attention to salient events engaging these circuits about 0.5 s earlier relative to activation by top-down attention. The contoured area denotes statistically significant canonical correlations with p < 0.005, and highlights that the overlap in bottom-up and top-down brain networks is slightly offset in time (mostly off the diagonal axis) with an earlier activation by salient events. A closer look at canonical vectors resulting from this correlation analysis reveals the topography of brain networks most contributing to this correlation. Canonical vectors reflect the set of weights applied to each voxel map that results in maximal correlation, and can therefore be represented in voxel space. These canonical vectors show a stable pattern over time lags of significant correlation and reveal a topography with strong contributions of frontal and parietal brain. Figure 5C shows a representative profile of overlapped canonical vectors obtained from SCCA analysis corresponding to the the time lag shown with an asterisk in Figure 5B and reveals the engagement of inferior/middle frontal gyrus (IFG/MFG) as well as the superior parietal lobule (SPL).

**Figure 6.**
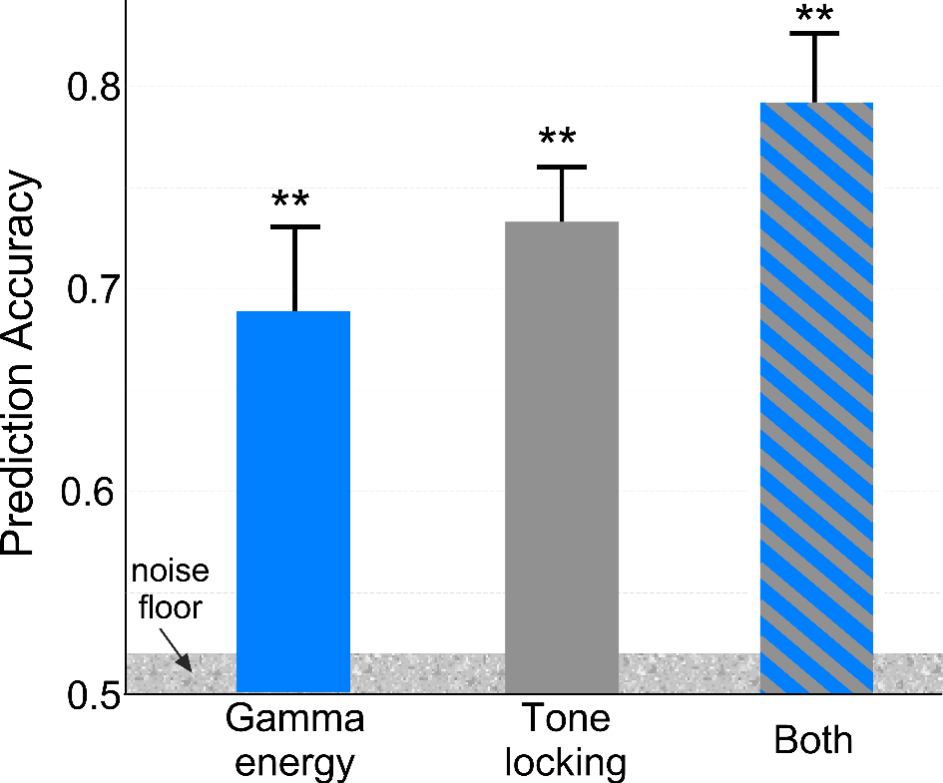
Event Prediction Accuracy. A neural network classifier is used to detect whether a tone in the attended sequence coincides with a salient event or not. The figure quantifies the average prediction accuracy (area under the ROC curve) resulting from training (and testing) the classifier using only high gamma band energy, only tone-locking energy, and both features. Error bars depict ±1 SEM. The noise floor is computed by shuffling feature values and labels (coincidence with salient tone).

Given the profound effects of bottom-up attention on neural responses, we examine the predictive power of changes in tone-locking and Gamma-energy modulations as biomarkers of auditory salience. We train a neural network classifier to infer whether a tone in the attended sequence is aligned with a salient distractor in the background scene or not. Figure 6 shows that classification accuracy for each neural marker, measured by the area under the ROC curve. Both Gamma and tone-locking yield significant predictions above chance [Gamma energy: 68.5% accuracy, t(9) = 4.12, p < 10^−3^; Tone-locking: 73% accuracy, t(9) = 6.03, p < 10^−5^]. Interestingly, the best accuracy is achieved when including both features [79% accuracy, t(9) = 7.20, p < 10^−7^], alluding to the fact that Gamma-band energy and phase-locking may contribute complementary information regarding the presence of attention-grabbing salient events in the background. Furthermore, an estimate of noise floor for this classification (see Materials and methods) yields a prediction range of 2% which is below the improvement in accuracy observed from combining both features. In addition, interaction information (IF) across these features was assessed. IF is an information theory metric that quantifies whether two features are complementary with respect to a class variable (Yeung, 1991; Matsuda, 2000; Shuai and Elhilali, 2014). This measure results in greater mutual information I(F1,F2;S)=0.65 using both gamma energy and tone-locking than the combination of both measures I(F1;S)+I(F2;S)=0.23+0.27, again suggesting a possible complimentary role of both features as biomarkers of salience.

## Discussion

Selective attention heavily modulates brain activity (Baluch and Itti, 2011; Knudsen, 2007), and bottom-up auditory attention is no exception (Ahveninen et al., 2013; Alho et al., 2014; Salmi et al., 2009). The current study reinforces the view that profound and dynamic changes to neural responses in both sensory and cognitive networks are induced by bottom-up auditory attention. It further demonstrates that these effects compete with top-down attention for neural resources, interrupting behavioral and neural encoding of the attended stimulus by engaging neural circuits that reflect the cognitive salience of ambient distractors. Modulation of both steady-state phase-locked activity in response to the attended stream as well as energy in the high gamma band is so profound that it can accurately identify moments in the neural response coinciding with these salient events with an accuracy of up to 79% relative to control -non salient- moments. The observed changes in both phase-locked activity as well as gamma oscillations dynamically change in *opposing directions* based on engagement of voluntary or stimulus-driven attention. This dichotomy strongly suggests shared limited resources devoted to tracking a sequence of interest, resulting in either enhancement or suppression of neural encoding of this attended target as a result of occasional competing objects (Lavie, 2005; Scalf et al., 2013). This push-pull action is strongly modulated by the salience of events in the ambient scene which not only reflect the dynamic acoustic profile of natural sounds, but also their higher-level perceptual and semantic representations, hence shedding light on dynamic reorienting mechanisms underlying the brain’s awareness of its surroundings in everyday social environments (Corbetta et al., 2008; Doherty et al., 2017). Further evidence of this push-pull interaction is also observed when accounting for subjects’ behavior, where trials with lower errors (suggesting higher attentional focus on the dynamic scene) result in higher enhancement of gamma power of targets and lower suppression by salient events; while trials with higher error (suggesting lower attentional focus) result in lower enhancement of targets and higher suppression by salient events.

The fidelity of the neural representation of an auditory stimulus can be easily quantified by the power of phase-locked responses to the driving rhythm. Enhancement of this phase-locking is accepted as one of the hallmarks of top-down attention and has been observed using a wide range of stimuli from simple tone sequences (similar to targets employed here) to complex signals (e.g. speech) (Elhilali et al., 2009; Mesgarani and Chang, 2012; Fuglsang et al., 2017). By enhancing neural encoding of voluntarily-attended sensory inputs relative to other objects in the scene, attentional feedback effectively facilitates selection and tracking of objects of interest and suppression of irrelevant sensory information (Knudsen, 2007; Shamma and Fritz, 2014). In the current study, we observe that diverting attention away from the attended stream does in fact suppress the power of phase-locking relative to a baseline level, tapered by the degree of salience of the distracting event (Figure 2C–D). The locus of this modulated phase-locked activity is generally consistent with core auditory cortex, though no precise topography can be localized from scalp activity only. Still, engagement of core sensory cortex in both enhancement and suppression of phase-locked responses to the attended sequence concurs with a role of auditory cortex in auditory scene analysis and auditory object formation (Ding and Simon, 2012; Leaver and Rauschecker, 2010). Moreover, the drop of steady-state following responses due to distractors coincides with a significant increase in the encoding of the background scene near highly-salient events, as reflected in the accuracy of the ambient scene reconstruction (Figure 3). Generally, the ability to decode entrainment to a dynamic envelope is a non-trivial task often yielding rather low reconstruction accuracy values (O'Sullivan et al., 2015; Vanthornhout et al., 2019); and is even more challenging for background sources away from attentional focus (Ding and Simon, 2012; Fuglsang et al., 2017). Still, neural decoding of ignored scenes results in boosted accuracy at specific moments coinciding with salient events. Such enhancement suggests that neural resources are indeed being diverted during those specific moments due to competition for neural representation between the attended target and the salient background object. Such diversion of resources is consistent with prior reports supporting load theory which posits that capacity limitations dictate the degree to which otherwise ignored sensory information can divert processing capability within and across modalities (Lavie, 2010; Salmi et al., 2009; Molloy et al., 2018).

Further analysis of this push-pull modulation of phase-locked activity rules out an interpretation based on acoustic masking which reduces the target-to-noise ration during distracting events resulting in a weaker auditory stimulation. A comparison of acoustic features throughout the background scene shows that there are no global differences between moments deemed attention-grabbing vs. background segments in the JHU DNSS dataset. Specifically, salient events were not confined to simply louder moments in the scene. Rather, some dense scenes contained ongoing raucous activity that would be perceived as continuously loud but not necessarily salient, except for specific conspicuous moments (e.g. emergence of a human voice or occasional discernible background music in an otherwise busy cafeteria scene). As such, what makes certain events salient is not the instantaneous acoustic profile independent of context. Instead, it is often a *relative* change in the scene statistics reflecting not only acoustic changes but also perceptual and semantic manifestations that normally emerge during everyday social settings. Moreover, excluding the loudest salient events (using both envelope-level and spectral-based measures) still reveals a significant drop of phase-locked activity relative to baseline tones further confirming that this effect cannot be simply attributed to energetic masking of the attended sequence. An additional analysis grouping salient events based on various low-level acoustic attributes (harmonicity, brightness) shows no difference in the degree of suppressed phase-locking induced by different sub-groups of events. Finally, the significant drop in behavioral accuracy in the attended task further confirms the distraction effect likely due to disengagement of attentional focus away from the attended sequence. It should however be noted that the behavioral design of the EEG experiment did not place any target tones in the post-event region in order to avoid any contamination of the neural signal. As such, it was not possible to perform a direct comparison of target tones and salient events and all analyses were compared against preceding time windows to assess the *relative* modulation of neural phase-locked activity as the stimulus sequence unfolded.

The engagement of executive attention resources in the current paradigm is further observed in enhancements in gamma-band activity, which is also consistent with prior effects linking modulation of gamma-band activity and engagement of cognitive control, particularly with attentional networks. Specifically, enhancement of high-frequency gamma has been reported in conjunction with top-down attention in auditory, visual, and somatosensory tasks (Debener et al., 2003; Tallon-Baudry et al., 2005; Bauer et al., 2006; Senkowski et al., 2005). While enhanced gamma oscillations index effective sensory processing due to voluntary attention, they also interface with mnemonic processes, particularly encoding of sensory information in short-term memory (Sederberg et al., 2003; Jensen et al., 2007). Given the demands of the current paradigm to remember the number of modulated targets in the attended sequence, it is not surprising to observe that the enhancement of gamma energy extends over a period of a few seconds post target onset, in line with previously reported effects of memory consolidation (Tallon-Baudry and Bertrand, 1999). In some instances, gamma band activity have also been reported to increase during attentional capture (Buschman and Miller, 2007); though two distinctions with the current study design are worth noting: First, our analysis is explicitly aligned to the attended target sequence hence probing distraction effects on activity *relative to* the foreground sound. Second, the modulation of gamma-band activity is very much tied with task demands, the notion of salience, and how it relates to both the perceptual and cognitive load imposed by the task at hand (Lavie, 2005). Importantly, the current paradigm emulates natural listening situations, which reaffirms the privileged status of social distractors previously reported in visual tasks (Doherty et al., 2017).

While effects of enhancement of broadband gamma frequency synchronization reflecting an interface of attentional and memory processing have been widely reported, reduction in gamma-band energy due to distraction effects is not commonly observed. Few studies, encompassing data from animal or human subjects, have shown a potential link between modulation of gamma energy and impaired attention, in line with results observed here. Ririe and colleagues reported reduced Local Field Potential activity (including gamma band energy) during audiovisual distraction in medial prefrontal cortex in freely behaving rats (Ririe et al., 2017). Bonnefond et al. used Magentoencephalography (MEG) in human listeners and reported reduced gamma for distractors during a memory task very much in line with observed effects in the current task (Bonnefond and Jensen, 2013). Moreover, modulation of gamma activity has been ruled out as being associated with novel unexpected stimuli (Debener et al., 2003) and is likely linked to ongoing shifts in attentional focus of listeners throughout the task reflecting the interaction between top-down executive control and sound-driven activity.

Importantly, the analysis of neural networks engaged during this push-pull effect points to overlapping networks spanning frontal and parietal areas involved during both bottom-up and top-down attention with a temporal offset for engagement of these networks (Figure 5). On the one hand, there is strong evidence in the literature dissociating dorsal and ventral networks of bottom-up and top-down attentional engagement, respectively (Corbetta and Shulman, 2002). Such networks have been reported across sensory tasks suggesting a supramodel attentional circuitry that interfaces with sensory networks in guiding selection and tracking of targets of interest while maintaining sensitivity to salient objects in a dynamic scene (Spagna et al., 2015). On the other hand, there are overlapping brain regions that underlie top-down as well as bottom-up attentional control particularly centered in the lateral prefrontal cortex, and interface between the two systems to guide behavioral responses that not only account for task-guided goals, but also environmentally-relevant stimuli (Asplund et al., 2010; Corbetta et al., 2008). Our analysis shows that there is a coordinated interaction between these two networks consistent with other reports of a possible shared circuitry, especially in the auditory modality (Alho et al., 2014). The current study specifically reveals a consistent temporal offset in the activation of the two networks. A possible interpretation of this dynamic interaction could be that signals from bottom-up attentional networks interrupt activity in the top-down network, potentially reorienting the locus of attention (Ahveninen et al., 2013; Corbetta et al., 2008; Salmi et al., 2009). Building on this interpretation, two possible hypotheses emerge from the earlier engagement of this common orienting network by bottom-up attention. One, that engagement of bottom-up attention sends an inhibitory reset signal in order to reorient attention to socially engaging salient events. Two, that earlier engagement of overlapped networks of attention by salient events reflects reduced memory consolidation due to a distraction effect, which is itself tied to diminished sensory encoding of the attended rhythm.

The ability to analyze the circuitry underlying the interaction between bottom-up and top-down networks in the current study was facilitated by a powerful multivariate regression technique named Canonical correlation analysis (CCA). This approach attempts to circumvent the limitations of comparisons across source topographies in electroencephalography (EEG) and leverages multivariate techniques to explore relationships between high-dimensional datasets which have been championed with great success in numerous applications including neuroimaging, pharmacological and genomic studies (Parkhomenko et al., 2009; Wang et al., 2015; Bilenko and Gallant, 2016). While applying CCA directly to very high-dimensional data can be challenging or uninterpretable, the use of kernel-based regularizations with constraints such as sparsity as adopted in the current work allows mappings between high-dimensional brain images (Witten et al., 2009; Gao et al., 2015; Uurtio et al., 2018). Here, we adapted the approach proposed by Rosa et al. (2015) as it not only regularizes the correlation analysis over sparse constraints, but also optimally defines these constraints in a data-driven fashion. The permutation-based approach yields an optimal way to define statistical significance of observed correlation effects as well as constraints on optimization parameters much in line with cross-validation tests performed in statistical analyses, hence reducing bias by the experimenter in defining parameters (Witten and Tibshirani, 2009; Lin et al., 2013). The resulting sCCA coefficients delimit brain regions with statistically significant common effects. Consistent with previous findings, the analysis does reveal that areas with statistically significant activations lie in inferior parietal and frontal cortices, particularly inferior and medial temporal gyri as well as the superior parietal lobule (Alho et al., 2014).

In conclusion, the use of a naturalistic experimental paradigm provides a range of nuanced ambient distractions similar to what one experiences in everyday life and demonstrates the dynamic competition for attentional resources between task and socially-relevant stimulus-driven cues. It not only sheds light on profound effects of both top-down and bottom-up attention in continuously shaping brain responses, but it also reveals a steady push-pull competition between these two systems for limited natural resources and active engagement by listeners.

## Materials and methods

### Stimuli

Auditory stimuli consisted of scenes from a previous study of auditory salience in natural scenes (Huang and Elhilali, 2017). This JHU DNSS (Dichotic Natural Salience Soundscapes) database includes twenty natural scenes, each roughly two minutes in length. All scenes were sampled at 22 kHz with a bit rate of 352 kbps, and converted to mono signals whenever applicable. Scenes were drawn from many sources (including Youtube, Freesound, and the BBC Sound Effects Library), and encompassed a wide range of sounds and scenarios. Some scenes were acoustically sparse, such as bowling sounds in an otherwise quiet bowling alley. Others were acoustically dense, such as an orchestra playing in a concert hall or a busy cafeteria.

Salience of events in each scene was measured using a dichotic listening behavioral paradigm (see [Huang and Elhilali, 2017] for full details). Human listeners were presented with two simultaneous scenes, one in each ear, and asked to indicate which scene they were attending to in a continuous fashion. The proportion of subjects attending to a scene compared to all other scenes was defined as salience. Peaks in the derivative of this salience measure were defined as salient events. The strength of salient events was further defined as a linear combination of the salience slope and salience maximum peak in a four second window after event. This salience strength was used to rank the events as high salience, mid salience, or low salience, with one-third of the events falling within each group. Each group contained 117 events, with a total of 351 across all scenes.

In the current study, each scene was presented one at a time, concurrently and binaurally with a sequence of repeating tones; together forming a single trial (Figure 1). The tone sequence consisted of repeated 440 Hz tones, each tone 300 ms long, with 10 ms cosine on and off ramps. Tones were presented at a rate of 2.5 Hz. A behavioral study was performed using Amazon’s Mechanical Turk to determine reasonable detection parameters for the tone sequence. Roughly 12.5% of the tones were amplitude modulated at 64 Hz to serve as targets for this behavioral modulation detection task; target tones were randomly positioned throughout the sequence. This experiment was developed using the jsPsych library (de Leeuw, 2015) and the Psiturk framework (Gureckis et al., 2016). Two modulation depths were tested: 0 dB (easy condition) and −5 dB (hard condition). During EEG recording sessions, tones were presented at a presentation rate of 2.6 Hz with a duration of 300 ms and 10 ms cosine on and off ramps. To avoid any confounds of neural effects between targets and salient events in background scenes, only three to five tones in each trial were amplitude modulated at 64 Hz with a modulation depth of 0 dB. Further, amplitude-modulated targets were constrained to be at least 1.5 s away from any salient event within the concurrent natural scene. A total of 79 modulated targets were present throughout the entire experiment. Stimuli (concurrent scene and tone sequence) were presented binaurally to both ears via a pair of ER-3A insert earphones.

### Participants and procedure

Eighty-one subjects (ages 22–60, twenty-seven female) participated in the behavioral study over Mechanical Turk. After a short training period, each subject performed ten trials, alternating between easy and hard conditions. The order of easy and hard conditions was counter-balanced across subjects. For each trial, a natural scene was presented concurrently with the tone sequence; subjects were instructed to devote their attention to the tone sequence and to press the space bar in response to each modulated target tone. Targets with a response between 200 and 800 ms after stimulus onset were considered hits; accuracy was calculated as the percentage of targets that were hits. In order to evaluate any distraction effects from salient events, we contrasted the detection accuracy of two groups of tones: (i) targets occurring between .25 and 1.25 s after an event (the period of the strongest event effect), (ii) targets occurring more than 4 s after any event (thus unlikely to be affected).

Twelve subjects (ages 18–28, nine female) with no reported history of hearing problems participated in the main EEG experiment. Subjects were tasked with counting the number of amplitude modulated tones within each sequence, and they reported this value at the end of the trial using a keyboard. Each subject heard each of the twenty scenes one time only. They were instructed to focus on the tone sequence and to treat the auditory scene as background noise. All experimental procedures (for both behavioral and EEG experiments) were approved by the Johns Hopkins University Homewood Institutional Review Board (IRB), and subjects were compensated for their participation. The sample size was powered to detect 30% difference in average phase-locking power to the stimulus rhythm between control (non-salient) and salient event epochs (power = 0.9, α=0.05).

### Electroencephalography

EEG measurements were obtained using a 128-electrode Biosemi Active Two system (Biosemi Inc, The Netherlands). Electrodes were placed at both left and right mastoids (for referencing) as well as below and lateral to each eye, in order to monitor eye blinks and movements. Data were initially sampled at 2048 Hz. 24 electrodes surrounding and including the Cz electrode were considered to be in the ‘Central’ area, while 22 electrodes surrounding and including the Fz electrode were considered to be in the ‘Frontal’ area (Shuai and Elhilali, 2014). Eight electrodes were located in the intersection of these two sets; all statistical tests were performed using the union of the Central and Frontal electrodes.

#### Preprocessing

EEG data were analyzed using MATLAB (Mathworks Inc, MA), with both FieldTrip (Oostenveld et al., 2011) and EEGLab (Delorme and Makeig, 2004) analysis tools. Neural signals were first demeaned and detrended, then highpass filtered (3rd order Butterworth filter with 0.5 Hz cutoff frequency). Signals were then downsampled to 256 Hz. Power line noise at 60 Hz was removed using the Cleanline plugin for EEGLab (Mullen, 2012). Outlier electrodes (around 2%) were removed by excluding channels exceeding 2.5 standard deviation of average energy in 20–40 Hz across all channels. Data were then re-referenced using a common average reference.

#### Data analysis

For event-based analyses (steady-state tone locking and gamma band energy), EEG signals were divided into epochs centered at the onset of a tone of interest (onset of modulated target, tone nearest a salient event, or onset of a control tone away from either target or salient event). Control tones were selected at random with the constraint that no target tone or salient event were contained within the control epoch; in total, 245 such control tones were selected. Each ‘epoch’ was ten seconds in duration (±5 s relative to onset). Noisy epochs were excluded using a joint probability criterion on the amplitude of the EEG data, which rejects trials with improbably high electrode amplitude, defined using both a local (single electrode) threshold of 6 standard deviations away from the mean, as well as a global (all electrodes) threshold of 2 standard deviations. Data were then decomposed using Independent Component Analysis (ICA). Components stereotypical of eyeblink artifacts were manually identified and removed by an experimenter.

For the tone-locking analysis, epochs were further segmented into 2.3 s (six tones) before and after onsets of interest (target, salient event, and control tone). All 2.3 s segments for a given group were concatenated before taking the Fourier Transform. This concatenation was performed to achieve a higher frequency resolution (given short signal duration and low frequency of interest). The concatenated signal was also zero padded to a length corresponding to 260 event windows in order to maintain the same frequency resolution for all conditions. As the analysis focused on spectral energy around 2.6 Hz, edge effects were minimal at that frequency region. Moreover, any concatenation effects affected all 3 conditions of interest equally. Finally, a normalized peak energy was obtained by dividing the energy at 2.6 Hz by the average energy at surrounding frequencies (between 2.55 and 2.65 Hz excluding the point at 2.6 Hz). The change in tone-locking power was defined as the normalized power using post-onset segments minus the power at pre-onset segments. For illustrative purposes, a tone-locking analysis was also performed over the full scene without dividing the data into epochs. (Figure 2A).

High gamma band analysis was also performed by taking the Fourier Transform of the data in two-second windows around attended targets, salient events, or control tones. The energy at each frequency and each electrode was normalized by dividing by the mean power across the entire event window after averaging across trials, and then converted to decibels (dB). The average power between 70 and 110 Hz was taken as the energy in the high gamma band. The change in gamma-band power was defined as the high gamma energy in the window containing 1.5 and 3.5 s post-onset minus high gamma energy between 2.5 and 0.5 s pre-onset.

All statistical tests that compared pre- and post-onset activity were performed using a paired t-test analysis. Effects were considered statistically significant if p≤0.05 and shown with ∗ in figures. Effects with p≤0.01 are shown with ∗∗. For non significant values, an additional Bayesian hypothesis test was conducted; a Bayes Factor below 0.33 provides good confidence that the null hypothesis is true (Dienes, 2014).

Events and tones were also divided into groups to test the influence of various conditions. Events were separated by salience strength into high, middle, and low salience events. Events were also divided based on low-level acoustic features, including loudness, harmonicity, and brightness (Huang and Elhilali, 2017). Finally, events and tones were divided based on the scene’s signed error, defined as number of target tones detected by a subject minus the number of target tones present during the scene. Positive signed error indicated high false positives while negative errors indicated high misses. A similar analysis was replicated using absolute error (—detected-actual— targets) with qualitatively similar results (data not shown).

### Envelope decoding

Decoding of the background scene envelope was performed by training a linear decoder, as described in O'Sullivan et al. (2015). Briefly, a set of weights (𝐖) was calculated to obtain an estimate of the envelope of the natural scenes ($\hat{𝐘}$) from the EEG data (𝐗) according to the equation $\hat{𝐘}=\mathrm{𝐗𝐖}$. Stimulus envelopes were extracted using an 8 Hz low-pass filter followed by a Hilbert transform. The EEG data itself was band-pass filtered between 2–8 Hz (both 4^th^ order Butterworth filters). Each linear decoder was trained to reconstruct the corresponding stimulus envelope from the EEG data, using time lags of up to 250 ms on all good electrodes. In contrast to the paper by O’Sullivan et al. which used least squares estimation, these weights 𝐖 were estimated using ridge regression (Fuglsang et al., 2017; Wong et al., 2018). Here, an additional regularization parameter (λ) is included to mitigate the effects of collinearity between independent variables (here the various EEG channels). The equation for estimating the weights is given by the equation $𝐖={\left({𝐗}^{T}\,𝐗+\lambda \,𝐈\right)}^{-1}\,{𝐗}^{T}\,𝐘$ (Wong et al., 2018). To avoid overfitting, a separate decoder was trained for each of the twenty scenes, using data from the remaining nineteen. The quality of these reconstructions was then evaluated over a range of regularization parameters λ by taking the correlation between the original stimulus envelope and the decoded envelope. A fixed high value of 2^20^ was chosen to maximize this overall correlation value for a majority of subjects. Finally, the correlation between estimated and original envelopes within a one second sliding window was calculated as a local measure of attention to the natural scene, and reported as reconstruction accuracy as shown in Figure 3.

### Gamma band source localization

Source localization was performed using the Brainstorm analysis software package for MATLAB (Tadel et al., 2011) and its implementation of sLORETA (standardized low resolution brain electromagnetic tomography). In contrast to other methods for converting electrode recordings into brain sources, sLORETA is a linear imaging method that is unbiased and has zero localization error (Pascual-Marqui, 2002). It is similar to minimum norm estimation, which minimizes the squared error of the estimate, but its estimation of source activities are standardized using the covariance of the data.

A surface head model was computed using the ICBM 152 brain template (Mazziotta et al., 2001) and standard positions for the BioSemi 128 electrode cap, in lieu of individual MRI scans. Gamma band activity was computed by first taking the Fourier Transform of the data in 500 ms windows with 200 ms step size centered around salient events, targets tones, and control areas, then averaging over frequency bands between 70 and 110 Hz. Energy at each voxel on the cortical surface was computed from the EEG gamma band activity using sLORETA, then z-score normalized across time for each trial.

### Voxel activation correlation

In order to compare brain networks engaged by bottom-up and top-down attention, we selected voxels of interest from the surface models during target tones $X_{T}\,\left(t\right)$, salient events $X_{S}\,\left(t\right)$, and control tones $X_{C}\,\left(t\right)$ for each subject. For each time instant t, target voxel activations (salient event activations, respectively) across trials were compared voxel-by-voxel to the control activations at the same relative point in time t using a paired t-test across all trials (significance at p=0.005). A Bonferroni correction was applied to select only voxels that were *uniquely* activated during target tones ${\hat{X}}_{T}\,\left(t\right)$ and salient events ${\hat{X}}_{S}\,\left(t\right)$ but not controls (Snedecor and Cochran, 1989). Using false discovery rates or random field theory to correct for multiple comparisons did not qualitatively change the final outcome of the correlation analysis (Hsu, 1996; Efron, 2010; Lindquist and Mejia, 2015). Based on this analysis, all voxels that were not significantly different from control activations were set to zero, therefore maintaining only voxels that were *uniquely* activated during target and near-event tones. As such, we are excluding shared activity that emerges from sensory response to tone presentations.

Next, matrices ${\hat{X}}_{T}\,\left(t\right)\,\left[n,v\right]$ and ${\hat{X}}_{S}\,\left(t\right)\,\left[n,v\right]$ representing unique activations across subjects n and voxels v for target and salient event responses at each time instant t were constructed, and columns of each matrix were standardized to 0-mean and 1-variance. A sparse canonical correlation analysis (CCA) was performed for each time index $\tau_{T}$ and $\tau_{S}$ to yield canonical vectors (or weights) $w_{T}$ and $w_{S}$ at times $\tau_{T}$ and $\tau_{S}$. CCA effectively determines linear transformations of ${\hat{X}}_{T}$ and ${\hat{X}}_{S}$ that are maximally correlated with each other following the objective function $ℒ:\max_{w_{S},w_{T}}q=w_{S}^{T}\,{\hat{X}}_{S}^{T}\,{\hat{X}}_{T}\,w_{T}$ (Uurtio et al., 2018). The weights $w_{T}$ and $w_{S}$ can be thought of as weights of the linear transformation from original voxel space that maximizes the correlation between the two datasets ${\hat{X}}_{T}$ and ${\hat{X}}_{S}$. Here, we specifically implemented a sparse version of CCA which optimizes the same objective function ℒ but imposes sparse, non-negative constraints on $w_{T}$ and $w_{S}$ using a least absolute shrinkage and selection operator (L1) penalty function, following the approach proposed in Rosa et al. (2015). This analysis resulted in a similarity measure across the entire dataset (for each time lag pair), while providing a combined set of sparse weights (canonical vectors) that can are easier to interpret in voxel space. A permutation-based approach was used to choose the regularization parameters. This technique performs a bootstrapping test across choices of regularization parameters by independently permuting data samples and maximizing correlation values across all data shufflings (Rosa et al., 2015). This same permutation also resulted in an overall significance metric for the final correlation values, by accepting correlation values q that are unlikely to be obtained through a random shuffling of the two datasets (Rosa et al., 2015). We performed the sparse-CCA across all time lags relative to targets and salient events in a cross-correlation fashion and scored as significant only the time lags that yielded a correlation value q with statistical significance less than p<0.005. For all time lags for which a statistically significant correlation was obtained, we visually compared the significant canonical vectors $w_{S}$ and $w_{T}$ which represent the sparse weights that yield a maximal correlation between the two sets and noted common activation areas.

### Predictions

Event prediction was performed using an artificial neural network. The analysis was performed on a per-tone basis where tones starting between 1 and 2.5 s before an event were labeled as ‘before’, while tones starting between 1 and 2.5 s following an event were labeled as ‘after’. Any tones that qualified for both groups due to proximity of two salient events were removed from the analysis. Two features were used in the classification: energy at the tone presentation frequency and gamma band energy. Both measures were averaged across central and frontal electrodes for each of the twelve subjects, resulting in twenty-four features used for classification. Gamma band energy was calculated by performing a Fourier transform on the data within a 2 s window around the onset of the tone, and then averaging energy between 70–110 Hz. A longer window was required to capture energy at the lower tone-presentation frequency. Thus, tone-locked energy was calculated by performing a Fourier transform on data within a 5 s window centered around the tone. Then energy at 2.6 Hz was normalized by dividing by neighboring frequencies (between 2.5 and 2.7 Hz). Classification was performed using a 3-layer feed-forward back-propagation neural network with sigmoid and relu activations for the first and second layers respectively and a softmax for the final layer (Deng, 2013; Goodfellow et al., 2016). The network was trained to classify each tone as either occurring before an event or after an event. Ten-fold cross-validation was performed by randomly dividing all of the events across scenes into ten equal portions. During each of the ten iterations, one group of events was used as test data and the remainder used as training data. A receiver operating characteristic (ROC) curve was constructed by applying varying thresholds on the network’s outputs and prediction accuracy was calculated as the area under the ROC curve. In order to compute a noise-floor for classification, three separate networks were trained, using gamma energy, phase-locking and both features combined consecutively, by shuffling the salience label of the neural marker and its correspondence near or far from a salient event. 10% of the shuffled data was used for testing yielding a random floor of predictions that reflects any underlying noise correlations in the data analysis. In a parallel analysis, mutual information between feature values ($F_{1}$: Gamma energy, $F_{2}$: tone locking) and salience label (S: near or far salient event) was computed. The metric quantifies interaction information I(F1,F2;S) which reflects whether two features are complementary with respect to a class variable (Yeung, 1991; Matsuda, 2000; Singha and Shenoy, 2018).

## Data Availability

Analysis from all data generated during this study are included in the manuscript and supporting files.

## Additional files

Transparent reporting form
