# Supplementary material for: Push-pull competition between bottom-up and top-down auditory attention to natural soundscapes
Source: eLife. 2020 Mar 20;9:e52984. doi: 10.7554/eLife.52984 (PMC7083598; doi:10.7554/eLife.52984)
Supplement: Peer review history

# Decision letter

**DOI:** 10.7554/eLife.52984.sa1

**Affiliations:** Peking UniversityChina; Université Claude Bernard - LyonFrance

In the interests of transparency, eLife publishes the most substantive revision requests and the accompanying author responses.

**Acceptance summary:**

Everyday life is full of switch between top-down and bottom-up attention. Although top-down attention has been widely studied, the neural mechanism for the saliency-based attention capture and their dynamic interplay, particularly in natural auditory scenes, remains largely unknown. This paper employed an elegant design by using a stream of natural sounds as background and examined how different levels of saliency in the background could modulate the neural response to foreground tones. They provide convincing evidence that the bottom-up and top-down auditory attention have opposite effects and compete for attentional resources.

**Decision letter after peer review:**

Thank you for submitting your article "Push-pull competition between bottom-up and top-down auditory attention to natural soundscapes" for consideration by *eLife*. Your article has been reviewed by three peer reviewers, one of whom is a member of our Board of Reviewing Editors, and the evaluation has been overseen by Barbara Shinn-Cunningham as the Senior Editor. The following individuals involved in review of your submission have agreed to reveal their identity: Nicolas Grimault (Reviewer #2).

The reviewers have discussed the reviews with one another and the Reviewing Editor has drafted this decision to help you prepare a revised submission.

Summary:

This study examined the competing process between top-down and bottom-up auditory attention and the neural mechanisms using a very interesting naturalistic approach. Several methodologies as behavioral, EEG and models are used jointly to build a strong and robust demonstration. The experiments are carefully designed, and the evidence is clear. Meanwhile, there are several major issues brought up by the reviewers that need the authors to address and do additional analysis.

Essential revisions:

1) About the conclusion regarding competition between bottom-up and top-down attention. The authors build their conclusion based on the enhancement and inhibition by top-down and bottom-up attention respectively. Meanwhile, the modulated target during top-down attention is still a transient event accompanying a physical change (amplitude modulation) and thus could still be interpreted as reflecting stimulus-driven, salient target instead of pure top-down attentional effect. Therefore, to claim the competition between bottom-up and top-down attention, the authors should justify that the responses to the modulated target are indeed indicative of processing related to top-down attention by new analysis or a new experiment.

2) About loudness control analysis. Loudness is a subjective measure and reflects a wide range of spectrum. Therefore, the analysis here is not a critical test for the contribution of energetic masking. It would be more appropriate to compare the phase-locking with the energy of the background around the tone frequency (e.g., 1-ERB around 440 Hz), instead of with loudness.

3) About low-level control analysis. Although the authors have performed control analysis to support that the saliency-induced effect is not due to loudness by excluding events with the highest loudness, this seems not enough to control low-level acoustic features. Could the authors use another low-level index, for example, the overall loudness, and did the same analysis to compare response to tones close to high loudness and tones close to low loudness? This would be a better control analysis to exclude at least loudness factors.

4) About the neural source analysis. The bottom-up and top-down attention has been previously posited to originate in dissociated brain networks, but here do the results suggest shared neural network with different temporal lag? Please add more clarifications.

# Reply: Author response

**DOI:** 10.7554/eLife.52984.sa2

Essential revisions:

1) About the conclusion regarding competition between bottom-up and top-down attention. The authors build their conclusion based on the enhancement and inhibition by top-down and bottom-up attention respectively. Meanwhile, the modulated target during top-down attention is still a transient event accompanying a physical change (amplitude modulation) and thus could still be interpreted as reflecting stimulus-driven, salient target instead of pure top-down attentional effect. Therefore, to claim the competition between bottom-up and top-down attention, the authors should justify that the responses to the modulated target are indeed indicative of processing related to top-down attention by new analysis or a new experiment.

In order to confirm that the neural response to targets is largely driven by top-down attention, we conducted a new analysis that focused on behavioral responses at the end of each scene. Although subjects do not make an immediate response to each modulated target, they do input the total number of targets they detected at the end of each trial, which can indicate whether or not they missed targets during the scene. Thus, for each subject, scenes were grouped by signed error, calculated as the number of targets reported by the subjects (detected targets) minus the number of targets in the scene (actual targets). A negative signed error, indicates that more actual targets were missed, suggesting that top-down attention was more distracted during these trials. Using signed error to breakdown responses with positive versus negative errors, we find that energy at both the 2.6 tone presentation frequency and gamma band energy exhibited higher increases after modulated target tones during the scenes with positive signed error than negative. The difference between the changes in gamma energy was highly significant [t(886) = 3.96, p = 8.06e-5]. The difference in tone-locking was present but not significant (t(886) = 0.73, p = 0.47), perhaps owing to reduced ability to isolate that specific frequency with too few epochs. Still, the strongly reduced effect in the gamma band with lower top-down attention clearly indicates that the effect is not solely a product of bottom-up processing of acoustic changes in the modulated tone. We replicated the same analysis using absolute error (absolute value of detected minus actual targets) and found qualitatively similar results (albeit at smaller statistical power, though still significant).

Along the same lines, we examined related aspects to the push-pull nature of the interaction between bottom and top-down attention by examining salient events in negative vs. positive signed error scenes. This analysis complements the results from top-down attention (discussed earlier) and focuses on effects of salience. In this analysis, we note that salient events in negative error scenes (more distraction) showed significantly higher increase in gamma than those in positive signed error cases [t(886) = 4.32, p = 1.74e-5]; suggesting that lower top-down attention indicated higher bottom-up attention, and vice versa.

Together, the increase/decrease in gamma energy with top-down/bottom-up attention with subjects’ behavior provides further support to a push-pull interaction between both forms of cognitive demands on subjects. These new analyses have been added to the text.

2) About loudness control analysis. Loudness is a subjective measure and reflects a wide range of spectrum. Therefore, the analysis here is not a critical test for the contribution of energetic masking. It would be more appropriate to compare the phase-locking with the energy of the background around the tone frequency (e.g., 1-ERB around 440 Hz), instead of with loudness.

As suggested by the reviewer, the phase-locking analysis was repeated after removing events with the highest energy in a range of 1-ERB around 440 Hz (calculated from Moore and Glasberg, 1983). After removing the events with the highest energy (top 25%) in that band, there was still a very significant drop in tone-locking following the events [t(443) = -4.93, p = 1.17e-6]. This new analysis is now included in the text.

3) About low-level control analysis. Although the authors have performed control analysis to support that the saliency-induced effect is not due to loudness by excluding events with the highest loudness, this seems not enough to control low-level acoustic features. Could the authors use another low-level index, for example, the overall loudness, and did the same analysis to compare response to tones close to high loudness and tones close to low loudness? This would be a better control analysis to exclude at least loudness factors.

A new analysis was conducted by splitting the events into two groups based on low-level acoustic features. Since loudness was quantified in two ways (using an overall measure and spectral based ERB-centered measure, see point #2 above), we explored additional low-level attributes. Harmonicity (how strongly pitched the sound is) and brightness (the centroid of the frequency spectrum) were examined since they were deemed important features based on their contribution to auditory salience (Huang and Elhilali, 2017). For both features, high and low events both had significant decreases in phase-locking and gamma power after events.

High harmonicity, phase-locking, [t(443) = -3.75, p = 1.97e-4]

Low harmonicity, phase-locking, [t(443) = -3.77, p = 1.82e-4]

High harmonicity, gamma energy, [t(443) = -3.61, p = 3.42e-4]

Low harmonicity, gamma energy, [t(443) = -7.68, p = 1.03e-13]

High brightness, phase-locking, [t(443) = -4.18, p = 3.51e-5]

Low brightness, phase-locking, [t(443) = -3.26, p = 1.21e-3]

High brightness, gamma energy, [t(443) = -9.30, p = 6.35e-19]

Low brightness, gamma energy, [t(443) = -2.99, p = 2.92e-3]

We added the results of this analysis on phase-locking to the text, in order to strengthen the claim that modulation of neural responses was not due to specific acoustic attributes.

4) About the neural source analysis. The bottom-up and top-down attention has been previously posited to originate in dissociated brain networks, but here do the results suggest shared neural network with different temporal lag? Please add more clarifications.

While there is indeed evidence in favor of distinct brain networks activated by top-down and bottom-up attention, a number of studies have already established some common activation areas from both forms of attention. For instance, Alho et al., 2015, have suggested that auditory attention may engage more overlap in bottom-up and top-down networks than visual attention, spanning temporal, parietal and frontal areas. This observation is in fact not unique to audition. Asplund et al., 2010, have used a visual paradigm and found convergent activations of stimulus driven and goal-directed attention in lateral prefrontal cortex. As such, the general observation of an overlapping activation is not surprising. The novelty with the SCCA analysis is to provide a better insight on the time lag of this common activation as well as pinpoint dynamics and span of this common activation. It is important to note that we confined the CCA to ‘correlated’ regions by design, after accounting for common activations due to the sensory drive (by removing overlap with control tones). Before applying CCA on voxel activations, we did observe that bottom-up attention elicited by salient events shows more activation in the lateral sulcus, presumably associated with primary auditory cortex; conversely, the top-down attention directed towards targets shows more activation in the superior parietal / frontal areas consistent with sustained attention. These are observations that were not included in the manuscript as we feel that proper statistical analyses are required in order to properly define these regions, which is not easily achieved with the current paradigm design and using EEG. Instead, SCCA offers the possibility of making statistically robust conclusions about underlying correlations in the data. We have edited the Discussion to better nuance the observation of existence of overlapped regions between bottom-up and top-down attention, as we feel this is an interesting conclusion that merits further work across modalities in order to better understand its implications.
